# Parents’ Attitudes Toward School Students’ Overuse of Smartphones and Its Detrimental Health Impacts: Qualitative Study

**DOI:** 10.2196/24196

**Published:** 2021-05-20

**Authors:** Ali Buabbas, Huda Hasan, Abrar Abdulmohsen Shehab

**Affiliations:** 1 Department of Community Medicine and Bahavioural Sciences, Faculty of Medicine Kuwait University Hawally Governorate Kuwait; 2 Department of Psychology, Faculty of Social Sciences Kuwait University Alshowaikh Kuwait; 3 Department of Immunology, Mubarak Al-Kabeer General Hospital Jabriya Kuwait

**Keywords:** smartphones, overuse impact, school students, parents’ attitudes

## Abstract

**Background:**

Parents’ awareness of the risks of the overuse of smartphones (SPs) among their children and parents’ attitudes toward this societal phenomenon are crucial factors to consider when investigating the causes and effects of, as well as interventions to control, this public health issue.

**Objective:**

This study aimed to explore the awareness and attitudes of parents regarding SP overuse among their children and the detrimental impacts associated with it.

**Methods:**

The qualitative method of semistructured face-to-face interviews was used to collect data from fathers and mothers of children aged 6-18 years from all 6 educational/governorate regions in the governmental sector in Kuwait.

**Results:**

A total of 120 parents agreed to participate in the study; there were more female (75/120, 62.5%) than male (45/120, 37.5%) respondents. Almost all of the participants (118/120, 98.3%) were aware that the overuse of SPs could lead to their children becoming addicted to the devices; they were also aware that there could be side effects on their children’s health (117/120, 97.5%). Although the participants, mostly the mothers, supervised their children’s use of SPs closely (106/120, 88.3%), the majority could not control their children’s length of time using SPs, as the children considered this a deprivation of their rights. Eye-related problems, headaches, and anger were the most common side effects experienced by the children.

**Conclusions:**

Although the parents were aware of the detrimental impacts of SP overuse, the majority could not control the length of time their children spent using the devices. It was found that strong social bonds among family members play a large role in controlling the use of SPs. A number of solutions for families and the government to combat the overuse of SPs are suggested.

## Introduction

### Background

Most of the early adopters of smart devices are from the younger generation, specifically teenagers [1]. Such devices have become an integral part of their lives, allowing them to stay connected with their friends and parents [2,3]. Smartphones (SPs) offer numerous advantages for users other than as mobile phones for communication: they can be used for playing games, watching videos, socializing via electronic media, and experiencing the array of information available on the World Wide Web. The widespread use of SPs has been reported worldwide, reaching 3.5 billion global users in 2020 [4], with South Korea reported to have the highest level of ownership of SPs [5]. Adolescent and elementary school students are, like adults, addicted to the use of SPs [6]. Pew Research Center reported that in 2019, 81% of Americans owned SPs [5] and nearly 95% of teens had access to SPs, and many of them had concerns about overusing them [7]. In Kuwait, according to a report on the consolidated Kuwait National Information and Communication Technology indicators, 99.5% of households owned SPs in 2019 [8].

The frequent use of SP devices for long periods of time can have an impact on users. Previous studies have shown that SP overuse is associated with physical health problems such as obesity; headaches; vision problems; and neck, shoulder, and back pain [8]. In addition, psychological problems have been identified, including anger and violence [9], loneliness and depression [10], and insomnia [11].

Furthermore, the overuse of SP devices can lead to addiction, especially among children and teenagers, who have weak self-control [12,13]. This population likes technology and uses it without awareness of the consequences. Regardless of the advantages of SP devices, the detrimental effects of their overuse are becoming apparent in society [2,14].

### Context

In the extant literature, most studies have used a quantitative approach to investigate parents’ perceptions of mobile technology use and its effects on their preschool children’s patterns of use [15,16], parents’ concerns [17], and parent-adolescent social relationships [2]. One previous study used a combined quantitative and qualitative approach to examine children’s routine behaviors regarding screen time from their parents’ perspectives and how the parents intervened to reduce the children’s sedentary lifestyle behavior [18].

There are crucial factors that contribute to the compulsive usage of SPs, including the user’s characteristics and experience. One study found that the more the user perceived enjoyment from using SPs, was satisfied with SP use, and liked using technology, the more they felt compelled to use SPs [19]. Another study found that perceived ease of use and perceived usefulness of SPs were factors that influenced behavioral intentions and thus social norms regarding the frequent use of SPs [20]. These factors are crucial aspects that cause SPs to play a prominent role in people’s lives.

In regard to children’s use of technology, a previous study aimed to identify strategies to control such usage [21]. In the study, 615 parents were surveyed and the results suggested that parents’ awareness about the negative impacts of long periods of screen time (>1 hour per session) and parents’ actions are the main requirements to regulate children’s use of technology [21]. A qualitative study was conducted in India using in-depth interviews to investigate parents’ opinions regarding their children’s use of mobile phones and how it affects their mental health [22]. The findings suggested that the unsupervised overuse of mobile phones among children could lead to mental changes, including stress [22].

Consequently, we are of the opinion that the views and experiences of parents are an important component to understanding the phenomenon of SP overuse among children [2]. There have been few studies on the awareness of parents of the risks of their children’s SP overuse and parents’ attitudes toward this societal phenomenon, which are considered crucial factors when investigating and determining which interventions to use to control this public health issue. The current study addresses the knowledge gap on this topic.

Therefore, this study aimed to understand the insights of parents in regard to SP device overuse among children of school age (aged 6 to 18 years). The objectives of this research were to (1) identify children’s patterns of SP use, (2) explore parents’ awareness of the detrimental impacts on health due to SP overuse, (3) identify parents’ attitudes toward the detrimental impacts associated with SP overuse, and (4) recommend appropriate interventions or solutions to avoid the risks to children’s health.

## Methods

### Study Design

A qualitative design employing semistructured face-to-face interviews was used to collect data from the parents (fathers or mothers) of school students. This is considered an effective approach in exploratory research to collect attitudinal information on a large scale to obtain in-depth information about specific phenomena [23,24].

### Recruitment and Data Collection

Data were collected from 120 parents of students from all 6 educational/governorate regions in the governmental sector in Kuwait: Asimah, Farwaniyah, Hawally, Jahra, Ahmadi, and Mubarak Al-Kabeer. Experts in qualitative research recommend that the optimal number of interviews should be between 12 and 60 [25]. Therefore, in this study, the data collection strategy was to interview 20 participants from each region to obtain data from different perspectives, as people from different regions can be expected to have different experiences and attitudes.

The schools were randomly selected from each educational region. The principal researcher contacted the schools’ managers to schedule the interviews during the parents’ meeting days. Parents were invited by the school managers to participate in this study, and those who agreed were taken to a quiet room next to the parents’ meeting hall. Only parents whose children used SP devices were included in this study.

At the beginning of each interview, the title and aim of the study were introduced to the parent. The average duration of the interviews was 25 minutes. The data collection process started in September 2018 and ended in May 2019.

The interviews were conducted by the principal researcher, who has skills in interviewing and knowledge of the research themes. This aided in standardizing the method of conducting the interviews, as the conditions of the interviews did not differ from one researcher to another.

### Face-to-Face Interview Guide

The interview questions were designed based on a review of the literature on related topics [2,17,18]. The interview guide aimed to achieve the objectives of the study (Textbox 1). It employed open-ended questions with probes to guide the interviews.

The interview guide was piloted with 5 parents (3 mothers and 2 fathers) to check the questions’ clarity, suitability for the study objectives, and order. Accordingly, minor amendments were made, which included adjusting the order of the questions and adding a question regarding the educational performance of the children to the interview guide. The interviews were conducted in Arabic because it is the official language in Kuwait; thereafter, the transcriptions were translated into English. The translations were performed by the translation office in the Faculty of Medicine at Kuwait University.

**The interview guide.**

**Demographic data**

- Participant’s age, gender, nationality, and educational level

**Students’ ownership of smartphone (SP) devices and patterns of use**

- The purpose of buying SP devices for your children: communication, entertainment, or education
- Your children’s patterns of SP device use: little use (only on the weekend or less than 2 hours/day), within moderate use range (2-4 hours/day), or overuse (more than 4 hours/day). The divisions of smart technology use were adapted from the Canadian Paediatric Society statement, where moderate use was defined as 2-4 hours/day [26]

**Level of awareness of parents of the detrimental impacts**

- The educational performance of your children and whether SP device use (ie, overuse) affects their performance: probes include “what is your child’s average grade?”
- Supervision of children’s SP device use: probes include close supervision, occasional supervision, or no supervision
- Awareness of the detrimental impacts (physical and/or mental) of overuse
- Physical health impacts (“have you noticed any of the following?”): seizures, nearsightedness, strabismus, dry eyes, blurry vision, transient blindness, headaches, sleep disturbance, neck/shoulder pain, lower-back pain, loss of concentration, or obesity
- Mental health impacts (“have you noticed any of the following?”): loneliness, anxiety, anger, depression, fear, annoyance, aggression, or lethargy

**Parents’ attitudes toward the overuse of SP devices**

- Reactions to the problem: start controlling the overuse, stop use (off/on), or arrange specialists to visit
- Overcoming this phenomenon: parental responsibility and governmental responsibility

### Ethical Considerations

Approval for the study was obtained from the Research Ethics Committee at the Kuwait Ministry of Health (reference number 885/2018). Parents’ consent was obtained prior to conducting the interviews, and parents were informed that they were free to withdraw from the study at any time.

### Qualitative Data Analysis

The interviews were audiotaped and transcribed verbatim. The transcripts were typed into Microsoft Word documents. A thematic analysis method was used to analyze the data because this simple qualitative approach can provide explicit results that are more understandable to the public [24,27]. In addition, this method is attractive to researchers because of its high flexibility of analysis. This method includes pinpointing, examining, and recording patterns or themes [27]. Initially, codes and subcodes were developed for the entire data set based on the themes of the semistructured interview guide. Then, an iterative approach comprising constant comparison was employed, in which all of the data relating to each theme was constantly revisited after the initial coding [28]. Reviewing and refining the themes and subthemes were done by the coauthors, in addition to cross-checking a random sample (n=12), to ensure consensus in the coding and the accuracy of the transcriptions. The data were entered into and analyzed using the software program MAXQDA Analytics Pro (VERBI Software GmbH), allowing the researchers to identify frequencies, compare themes, and find connections among the parents’ responses.

Four themes emerged from the analysis of the parent interviews: doctor’s advice, deprivation of the children’s rights, addiction to SP use, and the role of the government.

## Results

### Demographic Data

The total number of parents invited to take part in the study was 126; 120 of them agreed to participate, which provided a response rate of 95.2%. Twenty participants were interviewed from each region. Table 1 presents the demographic data of the interviewed parents. Among the interviewees, there were more mothers (75/120, 62.5%) than fathers (45/120, 37.5%), and more parents were Kuwaiti (104/120, 86.7%) than non-Kuwaiti (16/120, 13.3%). Most of the fathers (26/45, 57.8%) were in their 40s, and most of the mothers (41/75, 54.7%) were in their 30s. The majority of parents held a bachelor’s degree (fathers: 21/45, 46.6%; mothers: 49/75, 65.3%) or a diploma (fathers: 11/45, 24.4%; mothers: 17/75, 22.7%).

**Table 1:** Demographic data of the participants (N=120).

Characteristic			Educational region													Total, n (%)
			Ahmadi		Asimah		Farwaniyah		Jahra		Hawally		Mubarak Al-Kabeer			
**Gender**																
	Female	14		14		10		11		11		15		75 (62.5)		
	Male	6		6		10		9		9		5		45 (37.5)		
**Age group**																
	20-29	1		1		0		0		0		0		2 (1.7)		
	30-39	12		9		9		9		3		10		52 (43.3)		
	40-49	5		6		11		9		12		10		53 (44.2)		
	50-59	2		4		0		2		5		0		13 (10.8)		
**Nationality**																
	Kuwaiti	20		19		6		19		20		20		104 (86.7)		
	Non-Kuwaiti	0		1		14		1		0		0		16 (13.3)		
**Education level**																
	High school	2		1		3		3		1		3		13 (10.8)		
	Diploma	8		4		1		2		8		4		27 (22.5)		
	Bachelor’s degree	7		15		14		12		9		12		69 (57.5)		
	Postgraduate	3		0		2		3		2		1		11 (9.2)		

### Students’ SP Ownership and Pattern of Use

The majority of the participants (113/120, 94.2%) had bought SP devices for their children, while the minority (7/120, 5.8%) had given their children their own devices to use. The main reasons for their children using SPs were for entertainment (79/120, 65.9%), including playing games and watching videos on YouTube, and/or communication purposes (31/120, 25.8%).

The participants justified buying SPs for their children as imitating others (101/120, 84.2%) and keeping up in the era of technology (18/120, 15.0%). One parent stated,

Current society forces us to keep abreast with technology and imitate others in doing so...I bought smartphones for my children because their cousins had them.

a 32-year-old Kuwaiti mother of an 11-year-old girl, Mubarak Al-Kabeer region, interview number 11

More than half of the participants (68/120, 56.7%) declared that their children used SP devices for >4 hours/day, while 30.8% (37/120) said that their children used the devices for ≤4 hours/day. Some of the participants (15/120, 12.5%), of which 6.7% (1/15) were non-Kuwaitis, only allowed their children to use SP devices on the weekend, either with or without constraints on use:

I only allow my children to use smartphone devices at the weekend: it’s like a reward for them after five days of not using them, and they use them for more than six hours during the day—playing games, watching videos via the YouTube application and more…

a 43-year-old non-Kuwaiti father of an 8-year-old boy, Farwaniyah region, interview number 48

One mother described her worrying about her children when they were outside the house and her decision to let her children enjoy using SPs without constraints at home because at least they were around her:

I don’t mind allowing my children to have smartphone devices and use them for a long time if they are staying in the house. I worry about them when they are out and I don’t know where they are or whom they are with.

a 43-year-old Kuwaiti mother of a 13-year-old girl, Mubarak Al-Kabeer region, interview number 17

### Parents’ Awareness of the Detrimental Impacts of SP Overuse

The results revealed that the parents’ levels of awareness of the detrimental impacts of SP overuse were not associated with the interviewee’s age, gender, education level, or region. Almost all of the interviewed mothers and fathers were aware of children’s potential to become addicted to SP devices (118/120, 98.3%) and that there could be side effects as a result of SP overuse (117/120, 97.5%). One of the interviewees responded,

Yes, we know that using SP devices for a long time can lead to addiction to their use and also the side effects associated with overuse, and this information has been shared through social media.

a 45-year-old Kuwaiti father of a 15-year-old boy, Hawally region, interview number 89

When the participants were asked if the overuse of SP devices had negatively affected the educational performance of their children, 95.8% (115/120) responded with “no.” In fact, some of the parents had noticed improvements in their children’s educational performance. The majority (103/120, 85.8%) of the participants whose children were overusing SPs declared that their children had received final assessment levels of “very good” or “excellent” and sometimes showed better performance in English and general knowledge:

I have always tried to control my children’s use of smartphone devices, but I cannot do it—they still overuse them; however, their educational performance results are still the same or sometimes better.

a 39-year-old Kuwaiti mother of an 8-year-old boy, Asimah region, interview number 38

Another parent stated the following in an amazed way:

I have noticed that the English language of my son has improved, and I have realised that this is because of using SP applications and searching the internet.

a 33-year-old Kuwaiti mother of a 7-year-old boy, Jahra region, interview number 61

### Physical and Mental Health Problems

**Figure 1:**
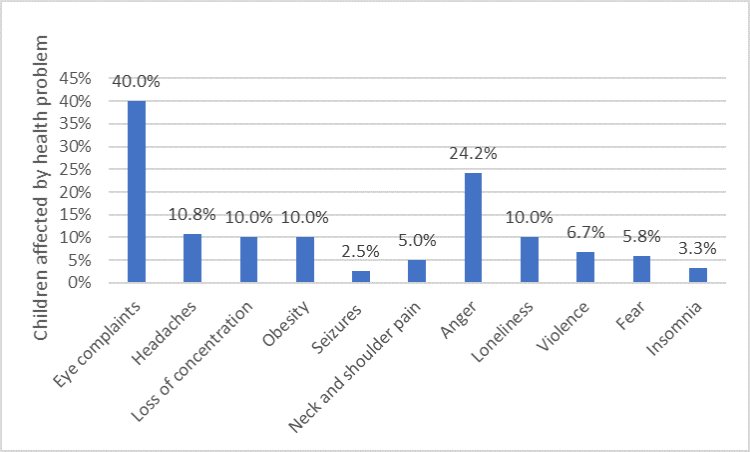
Reported physical and mental health problems in children due to excessive use of smartphones.

The results showed that almost one-half of the participants (56/120, 46.7%) had noticed specific health complaints among their children due to SP overuse (Figure 1), the majority of which were eye complaints (48/120, 40.0%), including eye dryness (16/120, 13.3%), blurry vision (15/120, 12.5%), and tired eyes (17/120, 14.2%). In addition, complaints related to the children’s mental state had been noticed (44/120, 36.7%) (Figure 1).

The results showed that some parents were distressed because their children often did not listen to their advice to play and socialize in “real life” and to reduce their online life with their SP. One parent expressed her dissatisfaction by noting the following:

...my daughter likes to stay alone in her room and most of the times she asks to bring the lunch and dinner meals to her room, and this is the cause of her obesity.

a 43-year-old Kuwaiti mother of a 13-year-old girl, Mubarak Al-Kabeer region, interview number 17

Furthermore, many of the parents reported that they had observed their children becoming angry or violent during or after SP use. Some of the parents reported that their children’s use of digital media via SPs had caused them fear and insomnia:

I realised that my son became scared and sometimes faced difficulty in sleeping...

a 36-year-old Kuwaiti mother of a 9-year-old boy, Jahra region, interview number 66

The participants’ responses revealed that the student’s age, gender, nationality, and educational region had no influence on his or her pattern of SP use and the physical and/or mental health complaints associated with it.

### Attitudes of Parents Toward Their Children’s Overuse of SPs

**Figure 2:**
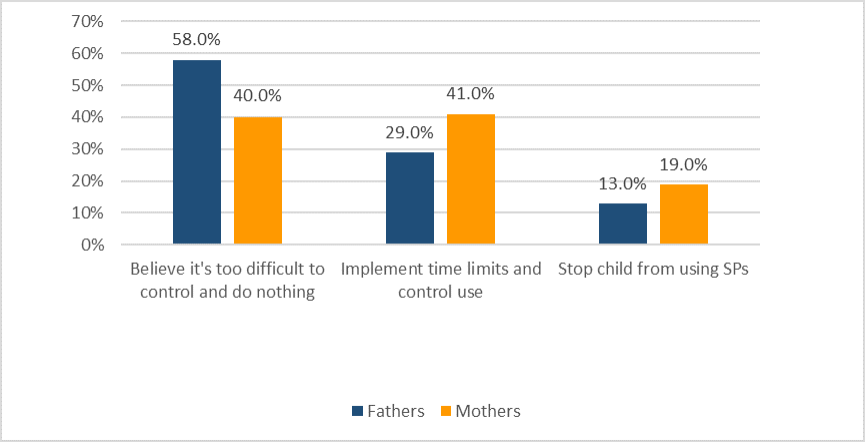
Parents’ reactions to their children’s overuse of smartphones (SPs).

Most of the participants (106/120, 88.3%), especially the mothers, were close to their children, supervised their SP use, and knew what their children were primarily using their devices for, such as accessing social media, communicating with friends, or playing games. When asked if they monitored their children’s patterns of SP use, most of the fathers (40/45, 88.9%) said that their wives were closer to their children than they were; however, because the couples shared the responsibility, fathers took over the role of monitor when their wives wanted to exercise more control over their children’s SP use. When parents noticed physical and/or mental health complaints in their children as a result of SP overuse, they showed different reactions; Figure 2 shows the different reactions of fathers and mothers. Among the non-Kuwaiti participants (16/120, 13.3%), half of them stated that SP use is necessary to keep abreast of developments in technology and that it is difficult to control SP use among children, while others believed in restricting the length of SP use. One participant’s response shows the difficulty of controlling children’s overuse of SPs:

To be honest, we tried many times to control the use of smartphone devices among our children, but we couldn’t because everybody uses them, even us...So, children feel that we deprive them of one of their rights.

a 39-year-old Kuwaiti mother of an 11-year-old girl, Mubarak Al-Kabeer region, interview number 15

Some parents showed good control over their children’s pattern of SP use, for which they identified a strong family bond as an important factor in the effective control of SP use. As one of the mothers stated,

...we are not only close to our children but also socialising with them and providing them with exciting alternatives to make them happy away from SP use...

a 42-year-old Kuwaiti mother of an 11-year-old girl, Mubarak Al-Kabeer region, interview number 7

The results also showed that doctors’ advice was important in encouraging parental firmness in controlling SP use among their children. One of the participants justified his reaction of stopping his child from using SPs as being because of a doctor’s advice:

Well, I am aware of the side effects of SP overuse, as my son has had brain seizures as a result of continuous overuse, so the physician advised us to stop using SPs, despite no one in the family having this symptom of epilepsy.

a 48-year-old Kuwaiti father of an 11-year-old boy, Jahra region, interview number 72

Another parent gave the following response:

...one of my cousins was addicted to SP device use, and, as a result, he had brain seizures; this made me very strict in controlling the usage time for my children, and I succeeded, as they got used to one hour a day...so we as parents need to be firm to save our children from harm.

a 42-year-old Kuwaiti mother of an 11-year-old girl, Mubarak Al-Kabeer region, interview number 7

Another parent had the following to say:

I know the negative effects of overusing SPs, especially among children, but, at the current time, I face difficulties in controlling their use among my adolescent children. It seems that we are waiting for something bad to happen to them to find a strong reason to stop them from using them...regrettably.

a 44-year-old Kuwaiti father of a 15-year-old boy, Hawally region, interview number 10

The results showed that the parents could not control the SP use of their children aged 15 years old and above, as they felt that their children were old enough to take responsibility for controlling their own SP use, which is a common behavior among adolescents.

### Parents’ Suggested Solutions

The parents were asked to suggest solutions to minimize the detrimental impacts of the overuse of SP devices on children (Tables 2 and 3). The most common solutions mentioned by the participants, with nationality having no influence, were implementing strict control in terms of allowing children specific times to use SP devices (fathers: 38/45, 84.4%; mothers: 67/75, 89.3%) and encouraging children to join health clubs and undertake sport activities (fathers: 23/45, 51.1%; mothers: 40/75, 53.3%). Other solutions were mentioned by a few participants: socializing as a family (fathers: 15/45, 33.3%; mothers: 27/75, 36.0%), encouraging participation in arts and science workshops (fathers: 4/45, 8.9%; mothers: 12/75, 16%), and using reward techniques (fathers: 6/45, 13.3%; mothers: 5/75, 6.7%).

**Table 2:** Suggested solutions from parents to minimize their children’s overuse of smartphone (SP) devices (N=120).

Suggested solution	Value, n (%)
Use strict parental control to restrict SP usage time.	105 (87.5)
Socialize as a family and go out for picnics, to farms, camping, etc	42 (35.0)
Encourage children to join health clubs and undertake sport activities.	63 (52.5)
Encourage children to participate in arts and science workshops.	16 (13.3)
Increase parents’ awareness of the fact that they are role models for their children.	15 (12.5)
Use reward techniques (eg, “If you study hard, you can use your SP for an hour”).	11 (9.2)
Block programs/games that have bad consequences with prolonged use.	5 (4.2)

**Table 3:** Suggested solutions from parents for the government to minimize the overuse of smartphone (SP) devices.

Suggested solution	Value, n (%)
Hold awareness sessions for school students on a regular basis, such as presentations by health specialists using examples of students who have suffered the detrimental effects of SP overuse.	64 (53.3)
Improve the awareness of parents, including methods to reduce their children’s SP overuse.	32 (26.7)
Monitor inappropriate programs for children and block them.	17 (14.2)
Use social media to provide advice and explain the detrimental impacts of SP overuse.	16 (13.3)
Arrange regular sports competitions for all ages in and outside schools for free and use famous players to increase participation rates.	18 (15.0)
Establish more sports clubs to accommodate more participants.	16 (13.3)
Reactivate science club activities.	3 (2.5)
Ensure computer classes at schools of all levels include lessons dealing specifically with the ideal use of SP devices, including recommended applications.	13 (10.8)
Establish an entertainment center in each region and arrange regular activities of all kinds throughout the year at minimal cost to attract participants of all ages.	15 (12.5)

The results showed that most of the participants (78/120, 65%) believed that it was not solely their responsibility to control the use of SP devices among their children but that the government also played a role. There were variances in the parents’ responses according to the educational/governorate region, with parents—specifically fathers—from Jahra and Ahmadi making more suggestions than participants from other regions regarding how the government could establish new sports clubs to accommodate more participants and large places nearby that would be suitable for family picnics.

Some of the participants (16/120, 13.3%) were frustrated and complained that they had noticed their children overusing SP devices but could not find useful alternatives:

The government has to support us as citizens in making an entertainment centre in each region, as well as establishing new sport clubs to accommodate more participants where currently they are incapable of doing so.

a 47-year-old Kuwaiti father of a 17-year-old boy, Ahmadi region, interview number 115

The results showed that the parents not only tried to offer advice to their children to reduce their overuse of SP devices but also gave them alternatives, as one of the respondents stated:

I have registered my children in a swimming course, and in their spare time I take them to a farm so that they can move freely without constraints.

a 48-year-old Kuwaiti father of an 11-year-old boy, Jahra region, interview number 72

In order to overcome the detrimental consequences of SP device overuse among students of different levels (primary, secondary, and high school), the majority of the participants suggested solutions (Table 2), and more than half of them indicated that the government also had a responsibility in this (Table 3). One of the parents declared,

Actually, there is a need to develop national programmes for education, training, and entertaining that involve activities throughout the year, aiming to attract the youth to spend their time in a productive way, and it’s very important to market these programmes smartly to ensure very good participation from all.

a 47-year-old Kuwaiti father of a 16-year-old boy, Jahra region, interview number 73

## Discussion

### Principal Findings

The findings of this study reveal that ownership of SPs among school students in Kuwait is high due to societal peer pressure, with people seeking to imitate one another. Such devices are mainly bought for entertainment and/or communication purposes, and partly for educational purposes. The majority of the parents were aware of the detrimental impacts of SP overuse; however, they expressed that it was difficult to control the SP overuse by their children.

### Children’s Patterns of SP Use

Most of the parents declared that their children’s use of SPs exceeded 4 hours on a daily basis, which is considered overuse by the American Academy of Pediatrics (AAP) and the Canadian Paediatric Society [26,29]. The parents admitted that they could not control their children’s duration of use of SP devices. Similar results in terms of parents worrying about SP device overuse and struggling to control the use by their children were also found in a previous study [30]. Furthermore, parents’ responses indicated a potential reason for their children’s persistent overuse of SPs: while parents might ask their children to reduce their use, they themselves overuse such devices in front of them, making controlling the use of SPs by their children difficult. This was reported in a previous study that found that children can be influenced by parental attitudes and beliefs; for instance, when parents were positive toward media use, their children used media for a longer time, and when parents were negative toward it, their children were deterred from using it as well [31].

### Awareness of the Detrimental Impacts of SP Overuse

Although almost all of the parents were aware that the overuse of SP devices could lead to addiction and other detrimental effects, including side effects related to physical and mental health problems, they also acknowledged that their children still used SPs heavily. It seems that parental awareness about the detrimental impacts was not enough to reduce SP overuse among children. Therefore, proper parental education and action are needed, wherein they can learn and use a variety of strategies to reduce the SP overuse, such as restrictions on technology use [32]. The findings revealed that almost half of the interviewed parents declared that their school-age children had suffered from numerous problems associated with SP overuse, including physical health problems: eye problems (tired, dry, and twitchy eyes), headaches, back and neck pain, difficulties in concentration, and brain seizures. These problems might be the result of staring at the screen of a small device for a long period of time and on a frequent basis, with strong light directed at the eyes. This association has been reported in previous studies in Saudi Arabia [33], Egypt [34], Turkey [35], India [36], and Poland [37]. In regard to brain seizures, for children who have been diagnosed with photosensitive epilepsy, the Epilepsy Society in the United Kingdom recommends avoiding the overuse of SP devices and reducing the frequent exposure to flashing and contrasting lights produced by the screens, which may trigger factors in the brain that cause abnormal nerve impulses and lead to convulsions [32]. Regardless of the strength of this association, it is crucial to know the causes behind students’ overuse of SP devices, which could be emotional, social, or other. Parents’ attention is required to solve the problem and reduce the overuse.

Furthermore, some of the parents reported an association between their children’s overuse of SPs and a sense of loneliness. More screen time, less movement, and fewer interactions with others can lead to depression and a sedentary lifestyle, which can cause obesity. This association could be because children need to play and socialize in real life, not just online, to feel connected to others [38]. Previous studies in Australia [39], Iceland [40], and China [41] have also reported that being less physically active and having more screen time are associated with depression. Interestingly, the participants in our study also believed that a sedentary lifestyle and excessive use of SP devices were associated with obesity, consistent with previous findings [18,42], and that the family environment plays an important role in this matter [18,43].

In this study, parents reported instances of their children becoming violent because of something pertaining to SP applications (such as challenging games) or angry while using social media or because they knew that their parents would stop their use at a specific time and they would be unable to continue to connect with the online world. This has also been reported in previous studies [10,12]. Some of the parents reported that their children’s use of digital media via SPs had caused them some fear and insomnia, and the parents realized that the content of the media determined the level of impact. The relationship between the use of mobile devices and poor sleep has been reported in several previous studies [44-46]. Therefore, it is of paramount importance that parents monitor their children to control their overuse of SPs in order to avoid physical or mental health problems.

The findings of this study revealed that the parents did not perceive their children’s overuse of SPs to be negatively impacting their educational performance, which was consistent with the findings of previous studies [47,48]. However, a study in Saudi Arabia concluded that medical students should decrease their SP use, as it was found to affect their academic achievement [33].

### Attitudes of Parents Toward Their Children’s Overuse of SPs

The results indicated that numerous parents were apathetic toward their children’s overuse of SPs, finding it too difficult to control. Children and adolescents typically have less self-control than adults and are easily distracted [12,13]. Smart technology, with its attractions and advantages for all ages, particularly teenagers, is often enjoyable. As technological applications develop and emerge, children come to depend on them and grow with them, resulting in a new generation with different health complaints, as this study shows. This was also consistent with a local study from Kuwait among school students, which showed similar health-related problems associated with SP overuse [45]. Most of the interviewed parents in this study stated that keeping abreast of technology is crucial but that the pattern of use must be well controlled to avoid harmful consequences. This makes good parental control of children’s use of SP devices important, especially during periods of behavioral development and physical growth, when parents play a vital role in taking care of them.

In the interviews, some of the participants revealed that when family bonds were strong, resulting in better socializing, there was good and effective control of SP use. Based on the parents’ responses, it appeared that not all of the parents were socializing with their children, but they showed a willingness to do so, believing it to be a good intervention to reduce the overuse of SPs. Previous studies have confirmed that good relationships between parents and children have a beneficial impact on children’s patterns of SP use [2,18].

Furthermore, some parents need physicians to advise them to take a firm and rational approach to their children’s SP use. One parent responded that he would probably implement a firmer approach to controlling his child’s SP use if his child developed a health problem, viewing health effects as a rationale for stopping the overuse of SPs. Parents and physicians should view a child’s visit to the physician’s office as an important opportunity to educate the child and parent regarding the possible detrimental health impacts of SP overuse.

Thus, leaving children to use SP devices without parental control leaves them susceptible to unknown risks that could expose them to physical and/or mental health problems. Hence, parents’ support via close supervision and participation with their children is of paramount importance for the safe use of SPs and healthy online participation [49]. Accordingly, the parents in this study suggested different solutions for families and the government to treat the problem of SP overuse, which should be viewed as a public health issue. In addition, the recommendations of the AAP [50] would be a very helpful resource for parents and schools in this regard. They suggest numerous ways to restrict smart technology use among children aged 0-18 years.

### Strengths and Limitations

The 2 main strengths of this study were as follows: (1) the sample of interviewed parents was large and included multiple perspectives from fathers and mothers, and (2) a high proportion of the participants were fathers (in many other studies, smaller proportions of the participants were fathers). On the other hand, this study had a number of limitations. First, it was limited to governmental sector schools, where the majority of students were Kuwaiti. Second, it only included parents, excluding their children from the study. Third, some of the questions asked the parents to recall their children’s health-related symptoms as a result of SP overuse, which could be subject to recall bias. Moreover, these health-related symptoms should not be attributed to SP use alone, as confounding factors were not accounted for because of the nature of the study. Fourth, due to the lack of research on similar populations in the region, most of the results of this study can only be compared with the findings of similar studies with populations from different cultures and environments.

### Conclusions

This study found that almost all of the participants, both fathers and mothers, were aware that the overuse of SPs could lead to addiction and other detrimental effects, such as physical and mental health problems. The parents were apathetic toward their children’s overuse of SPs, finding it too difficult to control. However, it was found that strong social bonds among family members could play a large role in controlling the use of SPs. It can be concluded that parents who provide a healthy family environment that encourages children to both socialize and play will support the children in avoiding the overuse of smart devices.

Based on the findings of this study, the following recommendations are suggested to avoid the detrimental impacts of SP overuse. First, parents should not only supervise their children’s SP use closely but also offer alternatives that help children enjoy their time away from online life. Second, although parents are generally aware of the health effects of SP overuse, they need training in cognitive and behavioral methods that can effectively improve their child’s self-control regarding SP use. Third, parents of a child who is overusing SP devices should consider a physician’s visit to ensure their child is free of its physical and psychological impacts and receive advice to help control their child’s SP use. Fourth, physicians need to be aware of the possible detrimental health impacts that SPs can have and to recognize their crucial professional role in this context, assisting in the development of local guidelines to address this matter. Fifth, the government should react to this public health issue and implement actions to meet the public’s needs for entertainment and sports facilities to provide alternatives to the use of SPs.

## Abbreviations

AAP: American Academy of Pediatrics
SP: smartphone
